# Supplementary material for: Longitudinal ultrasound assessment of jugular venous pressure reliably detects hypervolemia: An observational study in healthy volunteers
Source: PLoS One. 2026 Apr 9;21(4):e0346162. doi: 10.1371/journal.pone.0346162 (PMC13065051; doi:10.1371/journal.pone.0346162)
Supplement: Attachment — Submitted filename: Point-by-point answer.docx [file pone.0346162.s002.docx]

**Longitudinal ultrasound jugular venous pressure assessment shows high reproducibility and feasibility to detect hypervolemia – an observational study in healthy volunteers**

- **New title: Longitudinal ultrasound assessment of jugular venous pressure reliably detects hypervolemia: an observational study in healthy volunteers**

PONE-D-25-44241

Point-by-point answer

Dear Authors,
Thank you for submitting your manuscript to our journal. The objectives and the rationale are clearly reported. The manuscript structure and writing are well-organized and easily comprehensible.
The study design and limitations of the study are well described in the text.
This manuscript requires some adjustments. I kindly invite you to examine the suggestions and advice offered, especially concerning the statistical aspects.
Best regards,
VF Tripodi

Dear Professor Tripodi,

We sincerely thank you for your thoughtful feedback and positive evaluation of our manuscript. We appreciate your acknowledgment of our study’s clarity, structure, and design.

We have carefully considered the reviewers’ and your suggestions, particularly regarding the statistical aspects, and have made the necessary revisions to improve the manuscript accordingly. Detailed responses to each comment are provided in the attached revision report. Here you find the point-by-point answers addressing all comments and suggestions.

Best regards

Laura Potasso on behalf of all authors

**Review Comments to the Author**
Reviewer #1: Hello
This is a good article
Of course, I think you can make the title of the article shorter and more concise.
The text of the article is good, but it is better to collect and present the important points of the article in one place and in a few lines.
Good luck.

We sincerely thank the reviewer for this helpful suggestion. Following your advice, we have shortened the title to make it more concise and focused. In addition, we have summarized the most important findings of the study in a few lines at the end of the results section to provide a clear overview of the main results.

New Title:

Longitudinal ultrasound assessment of jugular venous pressure reliably detects hypervolemia: an observational study in healthy volunteers

New paragraph (lines 244-258):

**3.6 Summary of the results**

In our observational, methodological study of 30 healthy volunteers (50% female) comparing four uJVP methods, we found:

- **uJVP measurements:** All values were <8 cmH₂O, correctly below the hypervolemia threshold, whereas the hypovolemia threshold was exceeded in 56.7%, 21.4%, 51.7%, and 83.3% for methods 1–3b, respectively.
- **Best-performing method:** Longitudinal assessment at the base of the IJV tapering portion (method 3b) showed the highest interrater agreement (25/30, 83.3%) and could be visualized in 30/30 participants. Bland-Altmann analysis supported the use of Method 3b for consistent uJVP assessment between investigators (bias -0.25, limits of agreement -2.26 to 1.76). Acquisition time was 38.5 seconds, and tolerability (VAS 9.5) was comparable to other methods.
- **Practical recommendation:** Individualized head-of-bed elevation, visualizing uJVP in the mid-third of the neck, can help minimize confounding factors such as proximity to the subclavian vein or venous valves.

**Reviewer #2:**

The manuscript addresses an interesting and clinically relevant methodological question by assessing the reproducibility and feasibility of different ultrasound jugular venous pressure (uJVP) measurement techniques. The study is thoughtfully designed, clearly presented, and strengthened using a balanced, healthy volunteer population with blinded assessments. The authors are to be appreciated for their valuable contribution. With some refinements, this work has the potential to make a meaningful impact and be suitable for publication.

1. Correct the sequence of in-text citation references in the introduction (line 62), i.e., [1,2,12,4–11].
We thank the reviewer for pointing at this. We updated the references accordingly.

2. Statistical analysis (line 177 to 182)

I. The authors need to refine their choice of statistical tests. While the Wilcoxon rank-sum test is appropriate for comparing independent groups (e.g., male vs. female). Comparisons of uJVP values against expected values (one-sample data) would be more appropriately analyzed using a Wilcoxon signed-rank test. Similarly, when comparing different methods within the same patients (paired data), the Wilcoxon signed-rank test would be preferable.

Suggestion: For clarity, the authors could revise the statistical analysis section to indicate that both the Wilcoxon signed-rank test (for paired and one-sample comparisons) and the Wilcoxon rank-sum test (for independent groups) were applied as appropriate. This adjustment would improve the accuracy and transparency of the statistical analysis.

Thanks for this important suggestion. We revised the method section accordingly (lines 176-180):

We compared uJVP values to the predefined threshold using a one-sample Wilcoxon signed-rank test. Differences between methods within the same participants were assessed using a paired Wilcoxon signed-rank test. Sex differences in uJVP values were evaluated using the Wilcoxon rank-sum test (Mann–Whitney U test).

II. The authors need to reconsider how reproducibility is presented. Reporting it as the “percentage of participants with the same measurement within 1 cm” provides some indication of agreement but does not represent a standard reliability metric. More conventional approaches, such as intraclass correlation coefficient (ICC) or Bland–Altman analysis, would provide a stronger and more robust evaluation of inter-observer agreement. If these analyses are not possible, it would be helpful to explain the rationale for selecting a 1 cm tolerance and to note the potential limitations of this approach.

Thank you for raising this important point. We have added the Bland-Altman analysis to better visualize and compare the results. The rationale for using the percentage-based method was that, although the Bland-Altman analysis showed that Method 3b had the narrowest limits of agreement, these limits were within ca. 2 cm, which we considered insufficiently precise for clinical use. Therefore, we retained the analysis using a 1 cm difference to quantify the proportion of participants whose measurements fell within this stricter range.

We added the Bland-Altmann analysis in the section methods (lines 183-185), results (lines 227-236) and discussion (lines 294-295) as well as Figure 4.

Figure 4 – Bland-Altmann analysis

Bland-Altman plots for methods 1 to 3b. uJVP= ultrasound jugular venous pressure, LoA= limits of agreement.
