# Supplementary material for: Longitudinal ultrasound assessment of jugular venous pressure reliably detects hypervolemia: An observational study in healthy volunteers
Source: PLoS One. 2026 Apr 9;21(4):e0346162. doi: 10.1371/journal.pone.0346162 (PMC13065051; doi:10.1371/journal.pone.0346162)
Supplement: Attachment — Submitted filename: Point-by-point_2_Vogt.docx [file pone.0346162.s003.docx]

Point-by-point answer PONE-D-25-44241R1

**Longitudinal ultrasound assessment of jugular venous pressure reliably detects hypervolemia: an observational study in healthy volunteers**

Karin Vogt, David Widmer, Mark Kirsch, Laura Potasso

**Review Comments to the Author
Reviewer #3:** Dear authors,
I appreciate the opportunity to review the manuscript titled "Longitudinal ultrasound assessment of jugular venous pressure reliably detects hypervolemia: an observational study in healthy volunteers".
The manuscript structure and writing are well-organized and easily comprehensible.
The topic is truly interesting, and the manuscript has been revised in accordance with the reviewers’ requests.

Thanks a lot for your positive feedback and for your previous suggestions we were happy to implement.

**Reviewer #4:** • Primary endpoint choice and interpretation—be explicit and justify it
What you used: “proportion of participants whose measurements differed >1 SD from the expected uJVP value (6.5 ± 1.5 cm H₂O).”

We thank the reviewer for giving us the opportunity to explain this better. We added the rationale of using this approach in the paragraph 2.4 Endpoints, lines 151-153 and 162-163:

The primary aim of the study was to assess the accuracy and reliability of the current used uJVP assessment methods by comparing the assessment results with the existing normal values reported in the literature […]. The null hypothesis stated that there would be no difference compared to the expected value, i.e. that all methods will be equally accurate in measuring uJVP.

• Reproducibility: add standard reliability metrics (ICC ± CI) and present complete Bland-Altman numbers

What you did: percent agreement within ±1 cm and Bland-Altman plots; you state method 3b bias -0.25 and LoA -2.26 to 1.76 and percentages within 1 cm for each method.
Why this is insufficient: percent within ±1 cm is informative but not a standard reliability metric; ICC (two-way random, absolute agreement) with 95% CI and a table of Bland-Altman bias and 95% limits of agreement (numerical values for all methods) are expected

We implemented the requested analyses and added them in the section methods (lines 193-195) and results (249-251), commenting them in the discussion (lines 313-314):

*Methods*: Two-way Intraclass Correlation Coefficient (ICC) with absolute agreement, 95% confidence intervals (CI) and p-value were reported for all methods.

*ICC results (added in the manuscript in text)*: ICC with absolute agreement was 0.32 (95% CI -0.031 to 0.61, p=0.04) for method 1; 0.45 (95% CI -0.12 to 0.83, p=0.06) for method 2; 0.130 (95% CI -0.24 to 0.46, p=0.24) for method 3a; and 0.49 (95% CI 0.17 to 0.72, p=0.002) for method 3b.

|  | n | ICC | p | 95% CI |
| --- | --- | --- | --- | --- |
| Method 1 | 30 | 0.324 | 0.036 | -0.031 to 0.607 |
| Method 2 | 14 | 0.449 | 0.062 | -0.117 to 0.830 |
| Method 3 a | 29 | 0.130 | 0.244 | -0.236 to 0.463 |
| Method 3 b | 30 | 0.495 | 0.002 | 0.169 to 0.723 |

n= available assessment pairs; ICC= intraclass correlation coefficient; CI= confidence interval

*Bland-Altmann analysis results* (added in the manuscript as Table 4, lines 247-248)

|  | Bias | SD | LoA lower | LoA upper |
| --- | --- | --- | --- | --- |
| Method 1 | -0.30 | 1.54 | -3.32 | 2.72 |
| Method 2 | -0.83 | 2.83 | -6.38 | 4.72 |
| Method 3 a | -0.42 | 1.96 | -4.26 | 3.43 |
| Method 3 b | -0.25 | 1.02 | -2.26 | 1.76 |

SD= standard deviation; LoA= limit of agreement

• Missing/ambiguous denominators — make “n/total” consistent and transparent
Issue: method 2 has only 14 successful visualizations; Table 3 reports results for method 2 with denominators that look inconsistent (e.g., 3/14 appears in snippet). The abstract and different parts of the manuscript show slightly different percentages for method 2 (e.g., 21.4% vs 28.6% appears in different places of the document snippets). These inconsistencies must be fixed.

We thank the reviewer for this comment. However, we believe that this reflects a misunderstanding rather than an inconsistency. Pairs of visualizations for method 2 were possible in 14 out of 30 participants. In all other cases, at least one of the two investigators was unable to visualize the point at which the IJV is completely collapsed throughout the entire respiratory cycle. Table 3 reports the primary endpoint based on the number of successful visualization pairs, which is 3/14 for method 2 (21.4%). The secondary endpoints *successful visualization* and *reproducibility* were calculated for the entire cohort, i.e., the number of successful visualizations divided by the total number of participants (in case of method 2 are 14/30, 47%) and the number of reproducible measurements (= measurements within 1 cm tolerance) divided by the total number of participants (in case of method 2 7/30, 23.3%). These numbers are also reported in the abstract. For greater clarity, we have added the raw numbers in the abstract (lines 30-36), explained how the percentages were calculated in the Methods section (lines 163-166 and 188-199), and updated the labels of the first row of Table 3 (lines 222-227).

*Abstract, section results:* uJVP differed >1SD in 17/30(56.7%), 3/14(21.4%), 14/29(51.7%) and 25/30available assessment pairs (83.3%) respectively. Reproducibility for methods 1-3b was 18/30(60.0%), 7/30(23.3%), 19/30(63.3%) and 25/30participants (83.3%). Assessment was feasible in 30/30participants (100%) for methods 1 and 3b, 29/30(97%) for method 3a and 14/30(47%) for method 2.

*Methods:* The percentage was calculated by dividing the number of participants whose measurements differed by >1 SD from the expected uJVP value by the number of participants for whom both investigators were able to measure uJVP (= number of available assessment pairs) and then multiplying the result by 100.[…] Reproducibility was calculated in percentage of participants having the same evaluation from the two independent investigators with a tolerance of maximum 1 cm, divided by the total number of participants. […] Feasibility was assessed on the base of a) percentage of participants in which the IJV-height visualization was possible as defined by the method, divided by the total number of participants; b) duration of each available assessment in seconds and c) acceptance of the different US-assessment protocols by all the participants based on VAS.

• Sample size calculation wording and assumptions need clarification

The sample size section says “alpha and beta error of 0.05 and a power of 95%” that wording is confusing (alpha = 0.05 is fine; beta = 0.05 corresponds to power = 95%, but writing “alpha and beta error of 0.05 and a power of 95%” is redundant/unclear). Also, the formula printed is not standardly presented (formatting/notation broken).

We thank the reviewer for pointing at this, making us avoiding useless redundancy. We changed the format and the wording accordingly (Lines 177-178).

$$N=(SD\beta error+ SD\alpha error )^2/ (population mean- mean of study population )^2$$

with alpha error of 0.05 and a power of 95%, 29 participants were needed to verify the hypothesis. We rounded it at 30 to preserve the 50% sex distribution

• Methodological suggestions

Report full Bland-Altman statistics for all methods (bias, SD, and lower/upper 95% LoA numeric values) in a table (not just plots). You already show 3b numbers; add the others.
Add ICC (with 95% CI) for each method (two-way random, absolute agreement)
Explain the rationale for 1-cm tolerance:

Following your suggestions, we added Table 4 as reported above, we modified Table 3 and captions (lines247-251 respective 222-227), and included in the Methods section information on which patients *Duration* and *VAS* have been calculated (lines 195-198):

Feasibility was assessed on the base of a) percentage of participants in which the IJV-height visualization was possible as defined by the method, divided by the total number of participants; b) duration of each available assessment in seconds and c) acceptance of the different US-assessment protocols by all the participants based on VAS.

• Line/figure comments

• Table 3: add a footnote explaining denominators for method 2 (only 14/30 visualized) and show for duration/VAS.

Modified Table 3 with caption (lines 222-227)

|  | Participants with uJVP > 1 SD from expected measurement n/available assessment pairs (%) | Reproducibility/ inter-observer agreement  n/total participants (%) | Successful visualization  n/total participants (%) | Duration (s)  Median (IQR) calculated for successful visualizations | Tolerability (VAS,  10 = best)  Median (IQR) calculated for all participants |
| --- | --- | --- | --- | --- | --- |
| Method 1 | 17/30 (56.7) | 18/30 (60) | 30/30 (100) | 29.5 (20.8-41.3) | 9.8 (8.6-10) |
| Method 2 | 3/14 (21.4) | 7/30 (23.3) | 14/30 (47) | 32.5 (17.0-45.0) | 9.5 (8.6-10) |
| Method 3a | 14/29 (51.7) | 19/30 (63.3) | 29/30 (97) | 38.5 (23.5-62.0) | 9.5 (9.0-10) |
| Method 3b | 25/30 (83.3) | 25/30 (83.3) | 30/30 (100) | 38.5 (23.5-62.0) | 9.5 (9.0-10) |

Results of the primary and secondary endpoints for the different methods. Primary endpoint percentages were calculated using available assessment pairs as the denominator; secondary endpoint percentages were calculated using the total number of participants. Median duration was calculated only for successful visualizations; median VAS was calculated for all participants

uJVP = ultrasound jugular venous pressure, SD = standard deviation, IQR= interquartile range, VAS = visual analogue scale.

• Figures 3 & 4: Label the axes with units (//), add sample sizes to boxplots and Bland-Altman plots, and include a dashed line at the +5 cm constant if you show raw IJV height.

Modified Figure 3 with caption (lines 229-231)

Fig 3: Ultrasound based Jugular Venous Pressure (uJVP) results

Box-plot showing uJVP-results according to method. y-axis: in cmH₂O; dotted lines= expected mean 6.5cmH₂O (red) ± 1 SD (orange) based on previous studies.

Modified Figure 4 with caption (lines 243-245)

Fig 4: Bland-Altmann analysis

Bland-Altman plots for methods 1 to 3b. Orange vertical line identifies 5 cmH_2_O.

uJVP= ultrasound jugular venous pressure, LoA= limits of agreement.

• Abstract: ensure the percentage reported for method 2 is the same as in the Results/Table (fix the 21.4% vs 28.6% inconsistency).

We checked all numbers for consistency as described above, thanks again.
